# Effects of twenty-eight days of resistance exercise while consuming the commercially available pre- and post-workout supplements, NO-Shotgun® and NO-Synthesize®, on body composition, muscle mass and performance, and clinical safety markers in men

**DOI:** 10.1186/1550-2783-8-S1-P24

**Published:** 2011-11-07

**Authors:** Mike Spillane, Neil Schwarz, Sarah Leddy, Tracie Correa, Melodie Minter, Victoria Longoria, Darryn Willoughby, FISSN

**Affiliations:** 1 Exercise and Biochemical Nutrition Lab, Department of Health, Human Performance, and Recreation. Baylor University, Waco, TX 76798, USA

## Background

This study determined the effects of 28 days of heavyresistance exercise combined with the pre- and post-workout nutritional supplements, NO-Shotgun^®^ and NO-Synthesize^®^ onbody composition, muscle strength and mass, markers of protein synthesis and satellite cell activation, and blood clinical safety markers.

## Methods

Nineteen non-resistance-trained males were baseline tested and then randomly assigned to participate in a group that engaged in a resistance training program (3 X 8-10-RM) 4 times/wk for 28 days while also ingesting 54 g/day of placebo maltodextrose(PLC) or 27 g/day of NO-Shotgun^®^and 27 g/day of NO-Synthesize^®^ (NOSS). For PLC, 27 g were ingested 30 min prior to exerciseand 27 g within 30 min following exercise. NOSS ingested 27 g of NO-Shotgun^®^ 30 min prior to exercise and 27 g/day NO-Synthesize^®^within 30 min following exercise.Immediately upon waking on non-training days, PLC ingested 27 g of the supplement, whereas NOSS ingested 27 g of NO-Synthesize^®^. On day 29, participants were subjected to follow-up testing. Data were analyzed with separate 2 x 2 ANOVA (p < 0.05).

## Results

For dietary intake, there were no significant differences in total calories/day (p = 0.129) or in the daily amount of protein (p = 0.216), carbohydrate (p = 0.106), and fat (p = 0.665) between groups. For total body mass, both groups increased with training (p = 0.01), but there was no difference between groups (p = 0.793). However, NOSS underwent significant improvements in fat mass (p = 0.226) and fat-free mass (p = 0.023) compared to PLC. Both groups significantly increased muscle strength with training; however, for bench press (p = 0.023) and leg press (p = 0.035) NOSS was significantly greater than PLC. Serum IGF-1 (p = 0.038) and HGF (p = 0.001) were significantly increased with training, but were not different between groups. Myofibrillar protein increased in both groups with training (p = 0.041), with NOSS being significantly greater than PLC (p = 0.050). The levels of Type I, IIA, and IIX MHC were increased in both groups with training; however, Type I (p = 0.013) and IIA (p = 0.05) were significantly greater in NOSS. Muscle c-met was increased with training for both groups (p = 0.030), but not different between groups (p = 0.496). For total DNA, there was no difference between groups (p = 0.322) and neither group was affected by training (p = 0.151). All of the myogenic regulatory factors were increased with training; however, NOSS was significantly greater than PLC for Myo-D (p = 0.038) and MRF-4 (p = 0.001). No significant differences were located for any of the whole blood and serum clinical chemistry markers (p > 0.05).

## Conclusions

When combined with heavy resistance training for 28 days, NO-Shotgun^®^ and NO-Synthesize^®^ ingested before and after exercise, respectively, significantly improved body composition and increased muscle mass and performance. In addition, this supplementation regimen didnot abnormally impact any of the clinical chemistry markers.

## Funding

This study was supported by a research grant from VPX, awarded to Baylor University.

